# Screening for the ancient polar bear mitochondrial genome reveals low integration of mitochondrial pseudogenes (*numts*) in bears

**DOI:** 10.1080/23802359.2017.1318673

**Published:** 2017-04-27

**Authors:** Fritjof Lammers, Axel Janke, Cornelia Rücklé, Vera Zizka, Maria A. Nilsson

**Affiliations:** a Senckenberg Biodiversity and Climate Research Centre (BiK-F), Frankfurt am Main, Germany;; b Institute for Ecology, Evolution and Diversity, Goethe University Frankfurt, Frankfurt am Main, Germany;; c Faculty of Biology, Aquatic Ecosystem Research, University of Duisburg-Essen, Essen, Germany

**Keywords:** Mitochondrial genome, *numt*, Ursidae, polar bear, panda, pseudogene

## Abstract

Phylogenetic analyses of nuclear and mitochondrial genomes indicate that polar bears captured the brown bear mitochondrial genome 160,000 years ago, leading to an extinction of the original polar bear mitochondrial genome. However, mitochondrial DNA occasionally integrates into the nuclear genome, forming pseudogenes called *numts* (nuclear mitochondrial integrations). Screening the polar bear genome identified only 13 *numts*. Genomic analyses of two additional ursine bears and giant panda indicate that all except one of the discovered *numts* entered the bear lineage at least 14 million years ago. However, short read genome assemblies might lead to an under-representation of *numts* or other repetitive sequences. Our findings suggest low integration rates of *numts* in bears and a loss of the original polar bear mitochondrial genome.

Polar and brown bears are two well-recognized species, which differ in their morphology and ecology (Nowak 1999). Recent research has shown that polar bears diverged from brown bears in the mid Pleistocene (Hailer et al. 2012; Liu et al. 2014), which is supported by the fossil record (Kurtén & Anderson 1980) (but see Miller et al. (2012) for earlier divergence estimates). Phylogenetic analyses of mitochondrial DNA (mtDNA) showed that polar bears appear to be nested inside the brown bear radiation and dated the emergence of polar bears around 160 thousand years ago (kya) (Lindqvist et al. 2010; Edwards et al. 2011; Hailer et al. 2012). The deviating mtDNA phylogeny can be explained by recurrent introgressive hybridization between female brown bears and male polar bears 160 kya, resulting in a mitochondrial capture event that replaced the original polar bear mitochondrial (mt) genome (Miller et al. 2012). As evident from the low genetic diversity among polar bears, population bottlenecks during interglacials likely led to a fixation of the introgressed brown bear mtDNA in the polar bear lineage and a loss of the original polar bear mtDNA.

Occasionally mt genome sequences are transferred to the nucleus, and are incorporated as pseudogenes called *numts* (*nu*clear sequence of *mit*ochondrial origin, pronounced ‘new-mite’) (Lopez et al. 1994, Rogers & Griffith-Jones 2012). *Numt* insertions occur via non-homologous end joining at double-strand breaks in the nuclear genome. In general, the genomic fraction of *numts* is less than 0.1% with the highest proportion found in plants and yeast (0.28%) (Hazkani-Covo et al. 2010). Several genome scale studies have discovered that the total copy number and sequence length of *numts* varies widely between mammalian species (Hazkani-Covo et al. 2010). For instance, only 49 copies, totalling 6 kilo base pairs (kb), are found in the rat (*Rattus norvegicus*) genome while 1859 copies (2093 kb) are found in opossum (*Monodelphis domestica*) (Hazkani-Covo et al. 2010). Three different processes can contribute to the differences in the number of *numts* between species: the frequency of mitochondrial transfer, the amount of integrations as well as the dynamics of insertion processes.

To date *numt* insertions have not been studied in representatives of the bear family (Ursidae). If indeed, at 160 kya, the polar bear mt genome was replaced by the brown bear mt genome, all polar bear *numts* that entered the nuclear genome between the divergence of both species (∼600 kya) and the mitochondrial capture event (∼160 kya) are genomic ‘fossils’ of the original polar bear mtDNA (Figure 1). We screened the polar bear genome for *numts* to reconstruct the ancient polar bear mt genome sequence. Although the rate of *numt* integration in the genome is generally low, polymorphic *numt* copies are known from the human population (Dayama et al. 2014), indicating that these polymorphisms have spread within a few hundred generations. Screening the polar bear genome sequence, using the mt genome found in extant polar bears, identified 64 putative *numts* totalling about 29 kb sequence. The identified *numt* sequences cover 62% of a bear mt genome, i.e. 38% of the mt genome did not contribute to the polar bear *numt* landscape. We focused our analyses on 22 *numts* that were longer than 200 base pairs (bp), which represent 57% of the mt genome and represent mainly the NADH1 to COII region. Both rRNA genes as well as NADH4 and NADH5 are only partially covered (Figure 2). Phylogenetic maximum likelihood analysis of the *numt* sequences, including homologous mitochondrial sequences of all living bear species and other carnivores, indicated that the *numts* are older than 14 million years (My) (Supplementary Data 1). Fourteen of the 22 identified *numts* form clusters consisting of two or three *numt* fragments localized on the same scaffold (Supplementary Table 2). For each cluster, the scaffold localization, the gene order and orientation suggest that these are eroded fragments from single longer ancient integrations. Merging the consecutive fragments in a cluster reduced the total number to 13 *numts* (Supplementary Table 2). Aligning the genomic polar bear *numt* loci to orthologous sequences from the giant panda (*Ailuropoda melanoleuca*) (Li et al. 2010a) revealed that 11 of the 13 *numts* are present in full length in the giant panda genome and thus integrated at least 14 My ago, i. e. before the giant panda lineage diverged, (Kumar et al. 2017). Locus 7 was only partially identified and locus 8 was not identified in the giant panda genome. The genomic distance between *numt* fragments in the polar bear genome matched approximately the distance of the same fragments when mapped to the mt genome (Supplementary Table 3). *Numts* were fragmented by interspersed transposable elements (loci 7, 9 and 10) or expanding short tandem repeats (loci 6 and 11). Some of these interspersed sequences were only found in polar bear or giant panda, causing different inter-fragment distances (Supplementary Table 3). A full LINE1-1_Ame transposable element was inserted in locus 10 in the giant panda genome. Nearly all identified polar bear *numt* sequences seem to have been inserted prior to the evolution of Ursidae and no *numts* representing ancestral or recent polar bear mtDNA have entered the nuclear genome (Figure 1). As an additional line of evidence for decreased *numt* integration in Ursidae, we analyzed whole-genome sequencing data of two additional bear species for polymorphic *numt* insertions using the structural variation (SV) caller Lumpy (Layer et al. 2014). Our SV analyses date the previously identified *numt* insertions to at least before the diversification of American black (*Ursus americanus*), brown and polar bear, which gives a minimum age of 3 My (Kumar et al. 2017). However, the comparative screening of the giant panda genome indicate a much older *numt* insertion for most of the loci.

**Figure 1.**
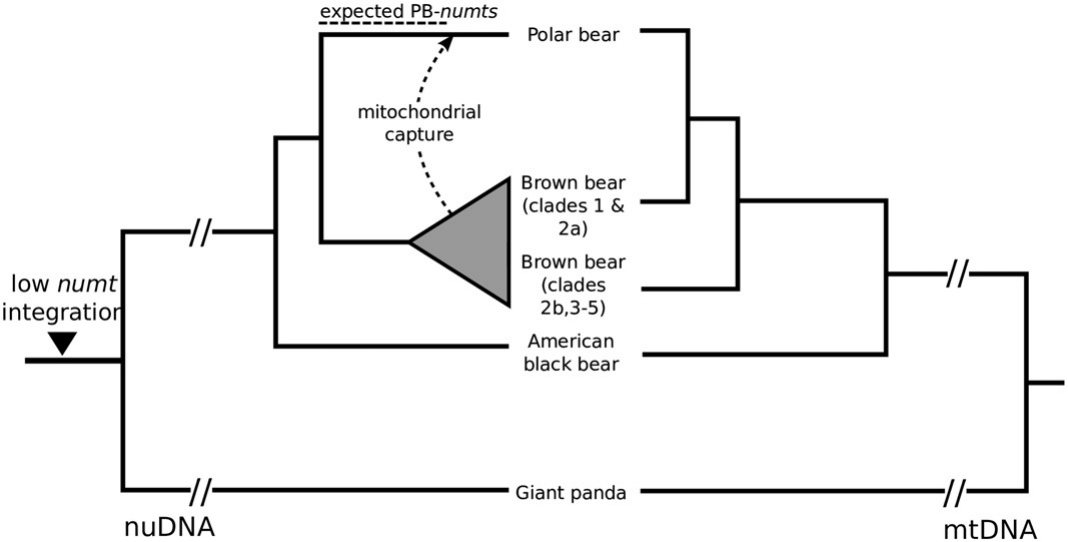
Phylogeny of bears reconstructed by nuclear DNA (nuDNA, left side) and mtDNA (right side). The left phylogeny reflects the speciation history of bears. About 160 kya, the original polar bear mtDNA lineage was replaced by brown bears (dashed arrow) causing the observed paraphyly of brown bears in the mtDNA phylogeny (right side). Dashed lines above the nuDNA phylogeny indicate the timeframe for potential integration of *numts* that represent the original polar bear mtDNA (PB-*numts*) and the observed reduction of *numt* integration in Ursidae.

**Figure 2.**
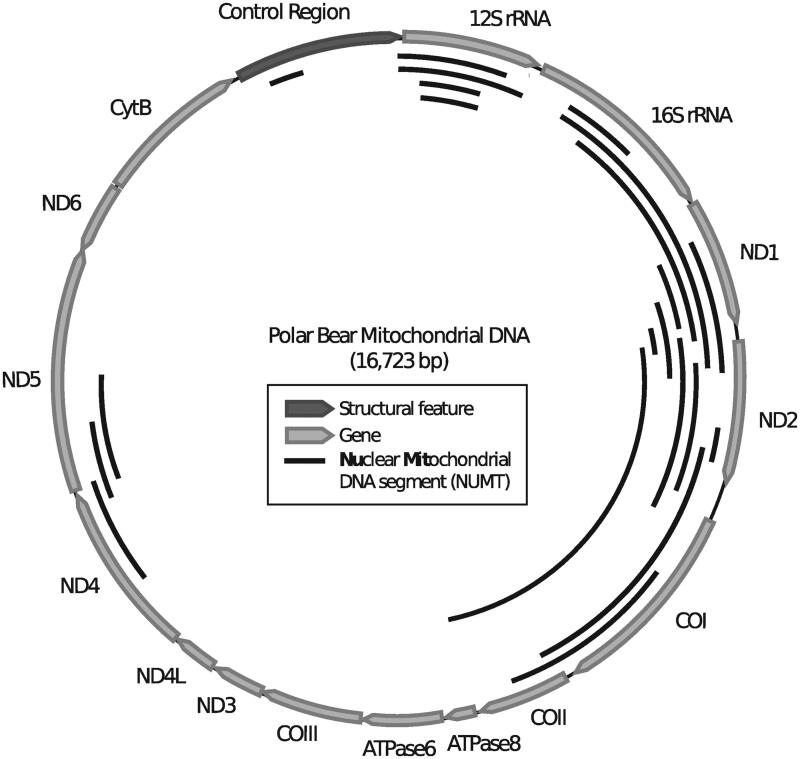
Genetic map of the polar bear mitochondrial genome with annotated genes. The identified *numts* longer than 200 bp are shown as solid black lines on the inside of the mt genome.

The number of *numts* and the genomic fraction derived from *numts* are not unreasonably low in the polar bear genome when compared to other mammals, that show similar low *numt* fractions. For example, the two murid rodents, mouse (*Mus musculus*) and rat only have between 6-39 kb *numts* in their respective genomes, which is among the lowest numbers of *numts* in mammals (Hazkani-Covo et al. 2010). The low incidence of *numts* in the closely related mouse and rat, as well as in the bear family suggest that these groups may have evolved mechanisms to suppress the integration of *numts* or that the rate of deletion is higher than in other groups (Hazkani-Covo et al. 2010).

However, another important reason for the lack of recent *numts* in the polar bear genome sequence may lie in the genome assembly processes. Recent *numts* are genetically similar to the mitochondrial genome, and de Bruijn graph based assembling algorithms might not be able to distinguish between short reads originating from *numts* or mtDNA (Li et al. 2010b; Hahn et al. 2013). This would cause a severe under-representation of recently integrated *numts* in short-read-based genome assemblies, and may in addition have introduced a bias in numerous published genome assemblies. So-called, third generation sequencing technologies like PacBio or Nanopore produce long reads, that are more likely to span complete *numt* insertion(s) and thus facilitate their incorporation into genome assemblies (Sohn & Nam 2016). To date, the gorilla genome is the only non-hominid mammalian genome generated by extensive usage of PacBio sequences (Gordon et al. 2016), however several additional long-read-based genome assemblies will likely become available in the next years. Thus, long-read-based sequencing of a bear genome can give further insights into the evolution of *numts* in Ursidae.

Recently inserted *numts* create problems for population and phylogenetic analyses of mt genes, if they are PCR amplified instead of the mt genes (Bensasson et al. 2001), but appear absent from the bear family. Thus, previous mitochondrial studies of bear phylogeny and population structures (Edwards et al. 2011) would be free from artefacts in the form of mitochondrial pseudogenes.

## Conclusions

The current polar bear genome assembly lacks recently inserted *numts*. The majority of the identified *numt* insertions have a minimum age of 14 My. A low *numt* insertion frequency has been reported for rodents and might be common in other mammalian groups. Thus, a low number of *numts* in bear genomes is not unreasonable, but an artefact from whole genome assembly algorithms cannot be excluded until further *de novo* assembled bear genomes become available. Utilizing long-read high-throughput technologies like PacBio or Nanopore might yield further insight into the fate of *numts* in Ursidae or other taxonomic groups with suspected *numt* depletion.

## Supplementary Material

TMDN_A_1318673_Supplementary_Information
