# Supplementary material for: Screening for the ancient polar bear mitochondrial genome reveals low integration of mitochondrial pseudogenes (numts) in bears
Source: Mitochondrial DNA B Resour. 2017 Apr 27;2(1):251–4. doi: 10.1080/23802359.2017.1318673 (PMC7894413; doi:10.1080/23802359.2017.1318673)
Supplement: TMDN_A_1318673_Supplementary_Information [file TMDN_A_1318673_SM2749.pdf]

Supplementary Information

Supplementary Data 1

Maximum likelihood trees of individual *numt* loci compared to mitochondrial orthologs of all bear species, dog and cat. Black numbers on branches indicate branch lengths and bootstrap values are given below branches (in red).

Supplementary Tables

Table S1: Accession numbers for complete mt genomes from Carnivora

**Description**	**Name**	**GenBank ID**
Polar bear	*Ursus maritimus*	AP012597
Brown bear	*Ursus arctos*	AP012592
American black bear	*Ursus americanus*	AF303109
Asian black bear	*Ursus thibetanus*	FM177759
Spectacled bear	*Tremarctos ornatus*	FM177764
Sun bear	*Helarctos malayanus*	FM177765.
Sloth bear	*Melursus ursinus*	FM177763
Panda	*Ailuropoda melanoleuca*	FM177761
Dog	*Canis lupus familiaris*	AB499817
Domestic cat	*Felis catus*	KP202275

Table S2: Genomic loci in the polar bear genome containing *numt* insertions. The number of *numt* fragments per locus (No. Fragments) and the locus length (Length) is given.

**Locus**	**Scaffold**	**Start**	**End**	**No. Fragments**	**Length**
1	scaffold1	19,176,679	19,177,582	1	903
2	scaffold100	5,568,310	5,570,656	1	2,346
3	scaffold126	3,684,606	3,684,896	1	290
4	scaffold159	746,082	746,875	1	793
5	scaffold18	12,418,188	12,419,257	1	1,069
6	scaffold18	19,909,859	19,919,849	2	9,990
7	scaffold299	350,193	357,102	3	6,909
8	scaffold39	17,871,509	17,875,385	1	3,876
9	scaffold449	24,328	30,629	3	6,301
10	scaffold46	296,529	307,643	3	11,114
11	scaffold55	2,339,127	2,341,674	2	2,547
12	scaffold615	1,384	4,336	2	2,952
13	scaffold73	4,946,867	4,947,069	1	202

Table S3: Clustered *numts* in the polar bear genome. For each clustered *numt* locus, the regions between fragments are labeled (a,b). The distance between the fragments on the polar bear mtDNA (Distance mtDNA) and the distance between fragments on the nuclear genome (Distance nuDNA) is shown. ΔDistance is the discrepancy between the distances on mtDNA and nuDNA. The presence of inserted transposable elements or short tandem repeats (STR) are indicated (Insert).

Locus No	Fragment gap	Distance mtDNA	Distance nuDNA	Δ Distance	Insert
6	a	6451	7761	1310	STR; L1* (frag)
7	a	724	927	203	SINE*
7	b	1718	1625	-93	
9	a	720	937	217	SINEC1D_CF*
9	b	3700	3406	-294	
10	a	2509	7137	4628	L1_Ame (frag)*
10	b	7629	9371	1742	L1_Ame*
11	a	102	554	452	STR
12	a	771	71	-700	

* Transposable element

Materials and Methods

BLAST search for numts in the polar bear genome

The mitochondrial genome sequence of the polar bear (AJ428577.1) was screened against the polar bear genome sequence (Liu et al. 2014) using BLAST (Altschul et al. 1990) with a word size of 20 bp to identify and localize insertions of mitochondrial DNA (*numts*) in the genome. The identified *numts* were filtered for length, and 22 *numt* hits longer than 200 bp were subjected to further analyses (equalling >90% of the cumulative numt length). *Numts* located within 10 kb distance to each other in the nuclear genome were merged to clusters, that consist of two to three fragments interspersed by genomic DNA.

Finding numts homologs in mitochondrial genomes of selected carnivore

The *numt* sequences from the polar bear genome were blasted against circularized (i.e. self-concatenated) mt genomes from all eight living bear species as well as dog (*Canis lupus familiaris*) and cat (*Felis catus*) (Supplementary Table 1) using word_size 20 and an E-value cutoff of 0.01.

Phylogenetic reconstruction of numt loci

The extracted polar bear *numt* sequences were aligned with mitochondrial homologs from Carnivora using MAFFT v7.305b (Katoh and Standley 2013) applying the --adjustdirection option.

Alignments were trimmed with trimal using “automated1” mode. RAxML 8.2.9 (Stamatakis 2014) calculated phylogenetic maximum likelihood trees using the GTRGAMMAI model. Node support was computed with 1000 bootstrap replicates.

Creating alignments of polar bear/giant panda and mitochondrial homologs

The clustered *numt* sequences plus 1 kb flanking sequences from the polar bear genome were queried against the giant panda sequence (Li, Fan, et al. 2010) using BLAT (Kent 2002). The list was sorted based on the alignment length and the ratio of BLATSCORE/ALIGNMENTLENGTH and manually screened for the extent of matching sequence between the polar bear *numt* loci and giant panda genomic sequence. The identified orthologs were aligned against polar bear sequences using MAFFT (Katoh and Standley 2013) and manually inspected. Using Aliview (Larsson et al. 2014), the resulting alignments were complemented with the previously identified mitochondrial homologs from the other carnivores. Repetitive element screening was performed using CENSOR (Kohany et al. 2006) and manually included in the alignment.

Whole-genome structural variation screen

For further investigation, whole-genome sequencing reads of two additional polar bear individuals (Accession no. SRR518686, SRR518687 and SRR518661,SRR518662) (Miller et al. 2012), one brown bear (SRR935592, SRR935595, SRR935624, SRR935628) (Liu et al. 2014), one American black bear (SRR518723) (Miller et al. 2012) were mapped against the polar bear genome as described elsewhere (Kumar et al. 2016). Lumpy (Layer et al. 2014) screened the genomes for structural variation using default settings. Predicted deletions longer than 200 bp were extracted from the SV dataset and tested for spatial association between genomic deletions of 100 bp and 10 kb in size and *numt* coordinates in the polar bear genome were investigated using bedtools multiinter (Quinlan and Hall 2010). Additionally, the short-read mapping was manually inspected using the Integrative Genome Viewer (Thorvaldsdóttir, Robinson, and Mesirov 2013).

Additional references

Altschul SF, Gish W, Miller W, Myers EW, Lipman DJ. 1990. Basic local alignment search tool. J Mol Biol. 215:403–410.

KatohK,StandleyDM.2013.MAFFTMultipleSequenceAlignment Software Version 7: improvements in performance and usability. Mol Biol Evol. 30:772–780. Kent WJ. 2002. BLAT-the BLAST-like alignment tool. Genome Res. 12:656–664.

KohanyO,GentlesAJ,HankusL,JurkaJ.2006.Annotation,submission and screening of repetitive elements in Repbase: Repbase submitter and censor. BMC Bioinformatics. 7:474

Larsson A. 2014. AliView: a fast and lightweight alignment viewer and editor for large datasets. Bioinformatics. 30:3276–3278.

Quinlan AR, Hall IM. 2010. BEDTools: a flexible suite of utilities for comparing genomic features. Bioinformatics. 26:841–842.

Stamatakis A. 2014. RAxML Version 8: a tool for phylogenetic analysis and post-analysis of large phylogenies. Bioinformatics. 30:1312–1313.

Thorvaldsdottir H, Robinson JT, Mesirov JP. 2013. Integrative genomics viewer (IGV): high-performance genomics data visualization and exploration. Brief Bioinformatics. 14:178–192.

Tsuji J, Frith MC, Tomii K, Horton P. 2012. Mammalian NUMT insertion is non-random. Nucleic Acids Res. 40:9073–9088.
